# Repeated Small Perturbation Approach Reveals Transcriptomic Steady States

**DOI:** 10.1371/journal.pone.0029241

**Published:** 2011-12-15

**Authors:** Ching-Lung Huang, Wun-Yi Shu, Min-Lung Tsai, Chi-Shiun Chiang, Cheng-Wei Chang, Chiu-Ting Chang, Ian C. Hsu

**Affiliations:** 1 Department of Biomedical Engineering and Environmental Sciences, National Tsing Hua University, Hsinchu, Taiwan; 2 Institute of Statistics, National Tsing Hua University, Hsinchu, Taiwan; 3 Institute of Athletics, National Taiwan Sport University, Taichung, Taiwan; Memorial Sloan Kettering Cancer Center, United States of America

## Abstract

The study of biological systems dynamics requires elucidation of the transitions of steady states. A “small perturbation” approach can provide important information on the “steady state” of a biological system. In our experiments, small perturbations were generated by applying a series of repeating small doses of ultraviolet radiation to a human keratinocyte cell line, HaCaT. The biological response was assessed by monitoring the gene expression profiles using cDNA microarrays. Repeated small doses (10 J/m2) of ultraviolet B (UVB) exposure modulated the expression profiles of two groups of genes in opposite directions. The genes that were up-regulated have functions mainly associated with anti-proliferation/anti-mitogenesis/apoptosis, and the genes that were down-regulated were mainly related to proliferation/mitogenesis/anti-apoptosis. For both groups of genes, repetition of the small doses of UVB caused an immediate response followed by relaxation between successive small perturbations. This cyclic pattern was suppressed when large doses (233 or 582.5 J/m2) of UVB were applied. Our method and results contribute to a foundation for computational systems biology, which implicitly uses the concept of steady state.

## Introduction

Systems biology studies the dynamics of networks of interacting molecules in living organisms [1], [2]. According to the new paradigm for biomedical study proposed by Kitano [2], a systems framework for biology has at least four key properties: I) system structure, II) system dynamics, III) control method, and IV) design method. An adequate experimental method of studying biological systems dynamics, particularly the transitions of physiological states (defined [3], [4], [5] according to various factors such as amounts of metabolites corresponding to metabolic states or RNA expression profiles for transcriptional states), has not yet been developed. Beyond the physiological state, physiological robustness [2] is also an essential feature for life to be maintained. To maintain the physiological robustness, a variety of levels of robustness, including transcriptomic expression, are critical. This can be referred as the transcriptomic expression steady state.

To unravel the complex regulatory networks underlying a living organism, many systems approaches have been applied to biological model systems. In those studies, chemical treatment [6], radiation exposure [7], [8], and physical stresses [9], [10] were frequently used to investigate their corresponding biological effects. However, the stimuli commonly used to investigate state transitions are often so intense that they casue exaggerated results leading to irreversible transitions of biological states, thus obscuring the physiological responses that occur under normal conditions. Here we present a new method of studying systems dynamics using a small perturbation technique; we also experimentally demonstrated the existence of steady states at the transcription level. The concepts of small perturbation and steady state used here are adapted from quantum physics. We used small doses of UVB radiation as a source of small perturbations to explore the gene expression profiles of disturbed biological states in auto-transformed human keratinocytes (HaCaT) [11].

Following repeated small perturbations of 10 J/m^2^ UVB, two opposite classes of genes, one down-regulated and the other up-regulated, exhibited an immediate response followed by relaxation between successive small perturbations. When larger doses (233 or 582.5 J/m^2^) of UVB were applied, however, these genes exhibited prolonged down- or up-regulation without relaxation. A cyclic pattern of gene expression following repeated small perturbations indicates the existence of steady states. This cycle pattern is suppressed when large perturbations are applied. In our experiments, the functions of up-regulated genes were mainly associated with anti-proliferation, anti-mitogenesis, and apoptosis. On the other hand, down-regulated genes were mainly related to proliferation, mitogenesis, and anti-apoptosis.

In conclusion, this study provides experimental evidence for the concept of steady state at the transcription level and demonstrates the feasibility of using small perturbation approaches for investigating steady-state phenomena. This study could also contribute to a foundation for computational systems biology, which implicitly uses the concept of steady state.

## Results

### Overview of Microarray Measurement

**Figure 1:**
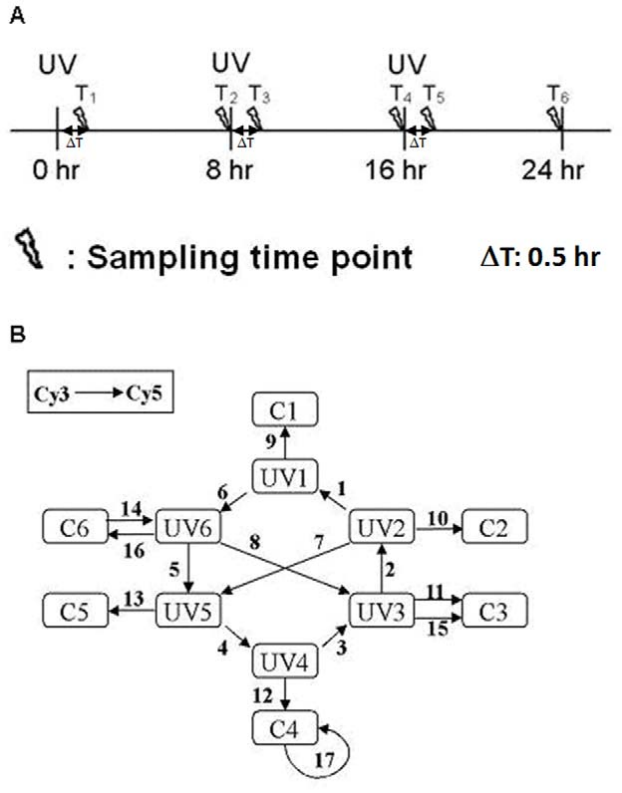
UVB stimulation and microarray experimental design. **A**) Three UVB exposures are indicated by UV at 0, 8, and 16 hours. T_1–6_ denote the sampling time points. T_1, 3, 5_ are timed 30 minutes after the corresponding UVB irradiation. T_2, 4, 6_ are timed 8 hours after each UVB irradiation. At each sampling time point, two samples (control and UV-irradiated) were collected. **B**) Seventeen dual-color microarray slides, each represented by an arrow, are used to measure the expression levels of the 12 samples. UVi/Ci denotes the UV-irradiated/control sample harvested at time T_i_. Samples appearing at the tail/head of an arrow are labelled with Cy3/Cy5. Slides no. 14 and 16 are dye-swap hybridizations. Slides no. 11 and 15 are technical replications. Slide no. 17 is a mock experiment.

We set up experiments (Figure 1A) to study gene expression profiles of HaCaT cells during multiple irradiations of UVB. The cells were irradiated at 0, 8, and 16 hours and harvested at 0.5, 8, 8.5, 16, 16.5, and 24 hours (T1 to T6 sampling time points) with its companion control. T1, T3, and T5 were timed to be 30 minutes after the corresponding UVB irradiation. T2, T4, and T6 were allocated 8 hours after each UVB irradiation and immediately before the next UVB irradiation (except T6).

One way to reduce the variance of microarray data is to increase the total number of measurements. Recent studies on microarray data processing suggested that loop design is more efficient than reference design [12], [13], [14]. Loop design (Figure 1B) was adapted and twelve samples were compared in this investigation. Samples C1 to C6 and UV1 to UV6 were non-irradiated and irradiated ones, respectively. Each sample was harvested at the indicated sampling time point.

In pre-processing the microarray data, we filtered out defective spots using the criteria described in Materials and Methods and then performed the loop calculation. In summary, 44% of spots (corresponding to 3.2 k genes) survived filtering. From the spots that survived, 2,019 genes were non-singular in the loop calculation. Among these 2,019 genes, we selected 155 genes with significant changes in gene expressions across the six sampling time points. For this selection, we set the standard deviation (SD) of the gene expressions at the six time points to be larger than 0.21 (see Text S1 for the selection criterion), which allowed 155 genes of the 2,019 genes to be submitted to the steady-state model-fitting algorithm (see Materials and Methods).

### Transcriptomic Steady State

**Figure 2:**
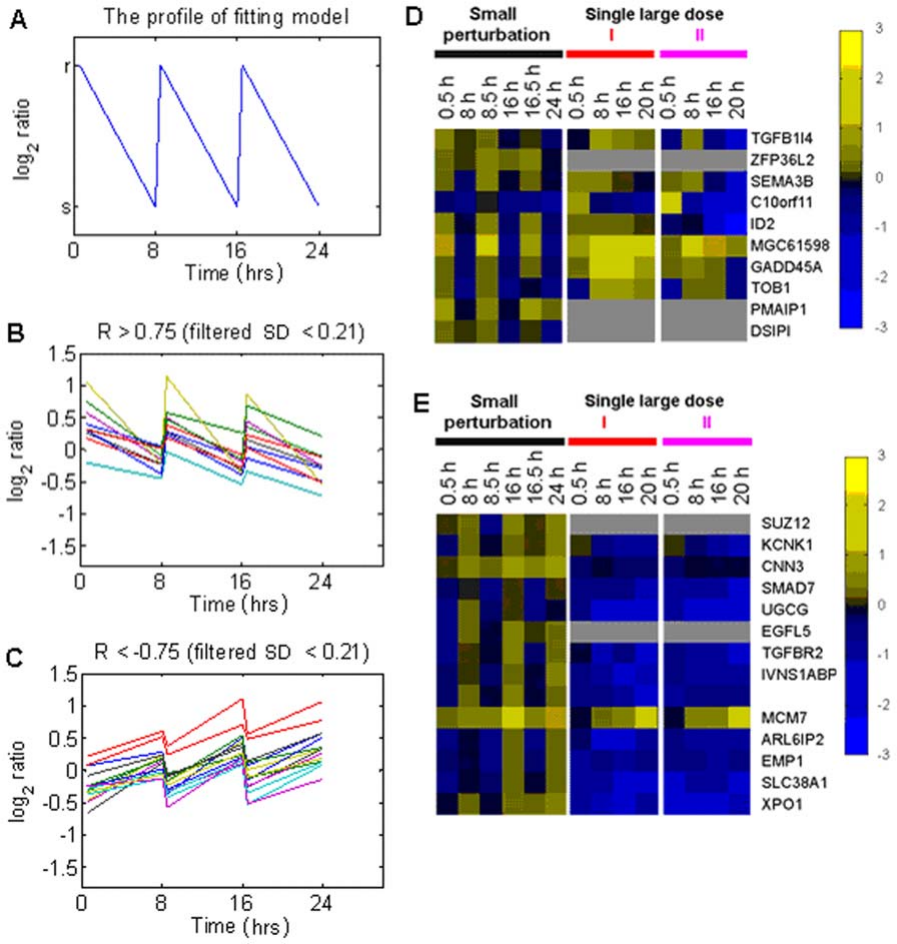
Results of steady-state model-fitting algorithm and the corresponding expression profiles of single large dose treatments. **A**) The profile of the fitting model. **r** and **s** are both arbitrary constants. Only genes with profiles of gene expression which changes significantly over time points (SD >0.21) were applied model-fitting algorithm. **B** and **C**) The expression profiles of genes with correlation coefficient, R, more than 0.75 (positive correlation) and less than −0.75 (negative correlation), respectively. Genes with correlation coefficient absolute value, |R|, greater than 0.75, as shown in (**B**) and (**C**) were considered to have a steady-state response. **D** and **E**) Steady-state genes that correlated positively (**D**) and negatively (**E**) with the model were randomly permuted in these two cases. Up and down regulation are presented by yellow and blue boxes, respectively; grey boxes indicate missing data. The gene expression profiles of the two groups of genes with small perturbation exhibit steady-state characteristics. For single large dose I (233 J/m^2^ of UVB) or single large dose II (582.5 J/m^2^ of UVB), the steady-state characteristics of the gene expression disappeared.

By applying the steady-state model-fitting algorithm to the expression data, genes with an absolute value of correlation coefficient, |R|, (Equation 2) greater than 0.75 (see Text S1 for the selection criterion) were considered to have a perturbation response followed by relaxation to steady state. For those genes with positive correlation coefficient (Figure 2B), their expression profiles are similar to that of the fitting model. For those genes with negative correlation coefficient (Figure 2A), their expression profile are opposite to that of the fitting model. Genes with a negative correlation (Figure 2C) were those whose expression profiles are opposite to that of the model. Two classes of genes with steady-state characteristics were identified by the steady-state model-fitting algorithm (Figure 2B, C and Table 1). Genes in class I were up-regulated and then relaxed (Figure 2B and D); genes in class II were down-regulated and then relaxed (Figure 2C and E) in response to repeated small UVB (10 J/m^2^) stimuli. Figure 2D and E also show expression profiles of steady-state genes after single large stimulus. Genes with a positive correlation coefficient in response to repeated small perturbations were almost consistently up-regulated at all time points (35 out of 56 time points, i.e., 62.5%) after both single large doses [I (233 J/m^2^) and II (582.5 J/m^2^)]. Genes with negative correlation showed an inverse pattern, i.e., almost all gene in this case were consistently down-regulated at all time points (88 out of 96 time points, i.e., 91.7%).

**Table 1:** The steady-state characteristic genes that tease out using the steady-state model-fitting algorithm are listed with gene name, referenced function(s), model classification, and corresponding source.

Model-fitting classification	Gene name	Functional annotation
up-regulated	TGFB1I4	proapoptosis [27]
up-regulated	ZFP36L2	development [28]
up-regulated	SEMA3B	proapoptosis [29]
up-regulated	C10orf11	unknown
up-regulated	ID2	proliferation [30]
up-regulated	MGC61598	unknown
up-regulated	GADD45A	cell cycle arrest, DNA repair, and cell death [31]
up-regulated	TOB1	antiproliferation [32]
up-regulated	PMAIP1	proapoptosis [33]
up-regulated	DSIPI	antiproliferation [34]
down-regulated	SUZ12	proliferation, antiapoptosis [35]
down-regulated	KCNK1	ion channel [36]
down-regulated	CNN3	cytoskeleton [37]
down-regulated	SMAD7	an antagonist of TGFβ signaling [38]
down-regulated	UGCG	differentiation, development [39]
down-regulated	EGFL5	development [40]
down-regulated	TGFBR2	TGFβ signaling [41]
down-regulated	IVNS1ABP	RNA splicing [42]
down-regulated		unknown
down-regulated	MCM7	DNA replication [43]
down-regulated	ARL6IP2	unknown
down-regulated	EMP1	proliferation, differentiation [44]
down-regulated	SLC38A1	glutamine transporter [45]
down-regulated	XPO1	nuclear export factor [46]

### Pathway Analysis of Genes with Steady-state Response

**Figure 3:**
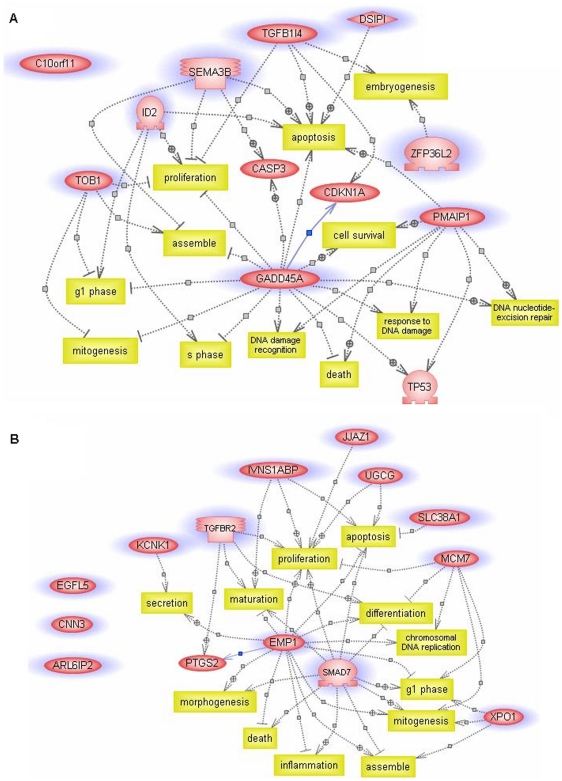
Pathway analysis of genes with steady-state response to repeated small UVB perturbations created using PathwayAssist. **A**) Up-regulated genes are primarily associated with the functions of anti-proliferation, anti-mitogenesis, and apoptosis. **B**) Down-regulated genes are mainly related to the functions of proliferation, mitogenesis, and anti-apoptosis. The plus and the minus symbols mark positive and negative regulations, respectively. Yellow boxes mark biological functions. Purple circles mark genes teased out by the steady-state model-fitting algorithm. Genes without data connection in the PathwayAssist database are pictured on the left without connection.

In order to gain insight into the relationships between those two classes of genes mentioned above, PathwayAssist (Stratagene, USA), a bioinformatics tool for identifying biological interactions among genes of interest from the published literature, was implemented. The functions of up-regulated genes are mainly associated with anti-proliferation (four out of ten genes), anti-mitogenesis (two out of ten genes), and apoptosis (six out of ten genes) (Figure 3A). On the other hand, down-regulated genes are mainly related to proliferation (six out of fourteen genes) and mitogenesis (four out of fourteen genes), and anti-apoptosis (two out of fourteen genes) (Figure 3B).

### Real-time PCR Confirmation

**Figure 4:**
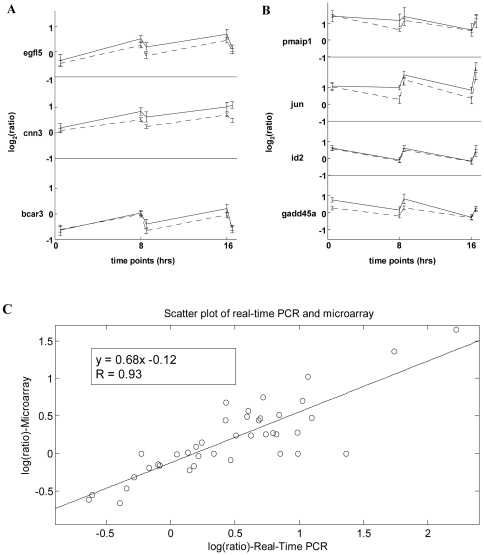
Comparison of microarray data with real-time PCR data. **A** and **B**) Seven genes were chosen randomly from the set of genes with steady-state characteristics for this comparison. egfl5, cnn3, and bcar3 (**A**) exhibit immediate down-regulated responses, while pmaip1, jun, id2, and gadd45a (**B**) exhibit immediate up-regulated responses followed by relaxation. Solid and dash lines represent real-time PCR data and microarray data, respectively. The error bar of the microarray data indicates standard deviation estimated by the log linear model. The error bar of real-time PCR data is the standard deviation derived from three sets of CTs. **C**) Scatter plot of real-time PCR results versus microarray data. The correlation coefficient, R is 0.93. The red line is a linear fit through all points. These results clearly demonstrate that even at the small fold change data points, these two sets of data agree very well. It also demonstrates the high reliability and resolution of our cDNA microarray system, which makes this kind of small perturbation study possible.

To confirm the gene expression changes observed by our cDNA microarray system, several critical genes were re-evaluated using real-time PCR (Figure 4A). The correlation coefficient R between results of cDNA microarray and those of real-time PCR of all tests is 0.93 (Figure 4B).

## Discussion

In this study, we used a new approach of repeated small perturbations to demonstrate the existence of a transcriptional steady state, which is a crucial step in life maintenance and an essential assumption of computational systems biology [15], [16], [17]. In performing this type of small perturbation experiment, timing and dosage are critical. In principle, different perturbation time periods would lead to the discovery of different sets of steady-state genes since recovery time—the time between perturbation and return to steady state—varies according to the type of gene. In order to induce detectable biological responses, the UVB doses used previously are usually higher than 80 J/m^2^ [18], [19], [20], [21]. However, such intense stimuli usually cause exaggerated results, leading to an irreversible transition of biological states. In contrast, perturbations that are too small may result in insignificant responses that are below the detection limit of the measurement systems used. Besides perturbation time periods, we believe that different doses of small perturbations would lead to the discovery of different sets of steady-state genes that in turn correspond to different physiological processes.

To the best of our knowledge, steady-state characteristics at the transcription level have not been systemically studied and experimentally proven. One important effect of this lack of experimental proof is that the external stimuli applied to biological models usually cause irreversible outcomes. Also, the high noise in gene expression profiles [22], [23] could hamper the detection of transcriptional steady state. Here, we adapted the concepts of small perturbation and steady state from quantum physics to the current study and experimentally proved the existence of transcriptomic steady states. A reliable cDNA microarray system, loop-designed statistical calculation, and computer modelling also played important roles in this study.

In this study, we demonstrated that repeated small-dose UVB exposure affects the expression of genes functionally related to proliferation and apoptosis in a cyclic pattern. In addition, for other groups of genes, cumulative effects following UVB exposure are evident. This may indicate that an eight-hour time period is not enough for these genes to relax to their basal level. Alternatively, the UVB dosage 10 J/m^2^ may be beyond the steady-state threshold for these genes. Therefore, whether a perturbation is sufficiently small could be depend on genes, pathways, and model organisms. From these observations, we conclude that experiments that use only one exposure duration and one dosage may be insufficient for revealing all UVB-regulated genes having steady-state phenomena. However, for the first time, we did observe that critical genes governing proliferation and apoptosis exhibit steady-state characteristics of gene expression.

The approach presented in this study provides a non-destructive means to investigate the biological black box. Therefore, in vivo experiments could be performed using this approach. Small perturbation experiments could potentially be applied to assess drug response by monitoring state transitions. Because such experiments better mimic real physiological responses, they can be used to reveal the mechanism of interest in a less ambiguous manner.

## Materials and Methods

### UVB Source and Irradiation

The light source was a 6 W UVB lamp assembled with a filter (EN-160, Spectronics Corporation, USA). The uniformity of the illumination field was well controlled. The coefficient of variation (CV) of intensities in the 55 cm^2^ illumination field was 2.15%. UVB dosage was measured with an IL-1400A radiometer (International light, USA) equipped with a wide band UV detector (SED005/WBS320/W, International light, USA). Before cells were exposed to UVB radiation, the culture media were collected in centrifuge tubes and cells were rinsed twice with pre-warmed PBS. HaCaT cells were then irradiated with 10 J/m^2^ (dose rate: 3.8 W/m^2^) UVB in 100 mm uncovered dishes (#430167, Corning, USA) with a thin layer of PBS at each indicated time point (Figure 1**A**). PBS was removed immediately after UVB irradiation. Cells were replenished with the previously collected medium and incubated at 37°C. A control set of cells was treated identically at each time point, except for UVB radiation.

### Microarray Fabrication

The microarray data of this work is MIAME compliant and has been deposited in GEO of NCBI (accession number: GSE7060).

Incyte Genomics supplied 9,600 human cDNA clones. After sequence verification, 7,334 clones were further amplified by PCR and purified by isopropanol precipitation in 96-well plates. The purified DNAs were re-suspended in 3X SSC for spotting. A single microarray slide (CMT-GAPsII, Corning Inc., USA) contains 7,334 human cDNA probes in quadruplicate, 10 spike-in genes (SpotReportTM-10 Array Validation System, Stratagene, USA) and one housekeeping gene, β-actin, in 96 replicates. Microarray slides were fabricated in a well-controlled environment (28±2°C and 48±1% humidity) and stored under desiccation until use. The arrayer system was assembled according to M-Guide (Patrick O. Brown laboratory, Stanford University, USA) and controlled by ArrayMaker version 2.5.1 (Joseph DeRisi laboratory, UCSF, USA) [24].

### Data Filtering and Normalization

The following three criteria were used to validate spots: 1) SNR [(signal-background)/SD of background] of Cy3 and Cy5 both greater than five, 2) Diameter of spot greater than 75 µm, and 3) CV (coefficient of variation) of pixels within a spot in the Cy3 and Cy5 channels both less than 100%. After this filtering process, on average about 45% of the spots were identified as valid.

We used MAANOVA [25], provided by Churchill's group, to perform normalization. To reduce the variation caused by printing pin and location on a slide, we used the within-print tip group normalization [26].

### Statistical Model of Microarray Loop Design

The normalized log ratios of the cDNAs were subsequently processed by using a log linear model (see Text S1). This algorithm assumes that the normalized log ratio of a clone is a sum of the three variables, γ, λ, and σ. These three variables are being evaluated for each clone. The variable γ is the dye bias correction of a clone among the sixteen arrays. The variable λ is an estimated relative expression level of a clone of twelve samples. The variable σ is the estimated value of random error of a clone among twelve samples. After the data were processed by the log linear model, a total of 2,019 genes are non-singular in the loop calculation.

### Steady-state Model-fitting Algorithm

In order to systematically tease out genes having characteristic steady-state expression patterns (quick response to the perturbation followed by relaxation), we developed a steady-state model-fitting algorithm. In the algorithm, the ideal pattern of a gene with steady-state response to the perturbation was defined as the vector v = (**r**,**s**,**r**,**s**,**r**,**s**), where **r** represents the response status to UVB irradiation and **s** represents the steady-state status (Figure 2A). At time point Ti, the response of a gene to UVB irradiation, denoted by xi, is the logarithm of ratio of the true expression level of UVi to that of Ci. We call the vector x = (x_1_,x_2_,x_3_,x_4_,x_5_,x_6_) the gene expression profile. Using the data from the 17 slides (Figure 1), the expression profile can be estimated via a log linear model and the least squares method (see Text S1). For a given expression profile, x = (x_1_,x_2_,x_3_,x_4_,x_5_,x_6_), of a gene, the correlation coefficient (R) between x and the ideal pattern v is
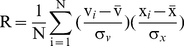
(1)where 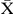 and 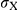 are the mean and the standard deviation of x, respectively. R can be deduced to
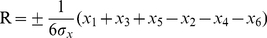
(2)


Its absolute value, |R|, is a measure of similarity between the expression profile x and the ideal steady-state pattern v. From Equation 1, which is equivalent to Equation 2, we see that R is the correlation coefficient of V and X. A correlation coefficient is always between −1 and 1. Figure 2 in the Text S1 shows the relative frequency histogram (red line) of the one million simulated, under the null hypothesis, R values and its smoothed approximation (blue line), f(x) = 0.75 (1−x^2^).

### Real-time PCR

The microarray data were verified by real-time PCR (TaqMan gene expression assays, Applied Biosystems, USA). All the real-time PCR results presented in this study were performed by an independent genomic core facility, National Research Program for Genomic Medicine, Taipei, Taiwan (http://genome.ym.edu.tw). Probes and primers for the interested genes (bcar3, cnn3, egfl5, gadd45a, id2, jun, and pmaip1) were chosen from the commercial database (Applied Biosystems, USA). The assay ID of corresponding probe sets and primers in the ABI's database are Hs00182488_m1, Hs00156565_m1, Hs00323519_m1, Hs00169255_m1, Hs00747379_m1, Hs01103582_s1, and Hs00382168_m1, respectively. All real-time PCR assays were performed in triplicate. Real-time PCR results were calculated by comparing UVB-irradiated experimental sets against control sets. Amplification of β-actin (ACTB, Hs99999903_m1) and glyceraldhyde-3-phosphate dehydrogenase (GAPDH, Hs99999905_m1) was used as an internal loading control for each individual amplification reaction. The quantity of target mRNAs was normalized to a housekeeping gene in each sample. Relative expression data are similar for two internal loading controls (β-actin and GAPDH) of real-time PCR. The results are shown in Figure 4 and only the data based on β-actin normalization are presented.

## Acknowledgments

The HaCaT cell line was kindly provided by Dr. Norbert Fusening (German Cancer Research Center, Heidelberg, Germany). We thank Genomic Medicine Research Core Laboratory, Chang Gung Memorial Hospital, Taiwan for the collaboration in fabrication of the cDNA microarray. The authors would like to thank Dr. Shu Chien, UCSD, for critically reading the manuscript and providing valuable comments.

## Supporting Information

Text S1 **Statistical model of microarray loop design.**
(PDF)
