# Supplementary material for: Repeated Small Perturbation Approach Reveals Transcriptomic Steady States
Source: PLoS One. 2011 Dec 15;6(12):e29241. doi: 10.1371/journal.pone.0029241 (PMC3240659; doi:10.1371/journal.pone.0029241)
Supplement: Text S1 — Statistical model of microarray loop design. (PDF) [file pone.0029241.s001.pdf]

Text S1 Statistical model of microarray loop design

Consider a two-color microarray experiment in which v samples (designated as “varieties” by some authors and called “target” in the context of hybridization) are compared using s slides. Each spot on a slide corresponds to a particular gene. We analyzed the data for each gene separately. With each spot on the slide in which samples k (labeled with Cy5) and k' (labeled with Cy3) are mixed and hybridized, there are two associated quantities, R and G, which are the normalization-corrected intensities of red (Cy5) and green (Cy3) dyes, respectively. We assume that the intensities are proportional to the true expression levels, denoted by μ_{kj} and $\mu_{k'j}$ for samples k and k', respectively, of the corresponding gene j. Since the data will be analyzed for each gene separately, for simplicity, we drop the suffix j in the following discussion and model R and G as follows:

$$R = r\mu_k\, 2^{\varepsilon^{(R)}},\ \ G=g\mu_{k'}2^{\varepsilon^{(G)}}$$

where r and g are the proportional factors of red and green dyes, respectively. $\varepsilon^{(R)}$ and $\varepsilon^{(G)}$ are random error terms. The logarithmic ratio is

$$\begin{aligned} Y &\equiv \log_2\left(R/G\right)=\log_2(r/g)+\log_2\left(\mu_k/\mu_{k'}\right)+\left(\varepsilon^{(R)}-\varepsilon^{(G)}\right) \\ &\equiv \gamma+\log_2\left(\mu_k/\mu_{k'}\right)+\varepsilon \end{aligned}$$

where the parameter $\gamma \equiv \log_2(r/g)$ represents the relative labelling efficiency between dyes. Let $\bar{\mu}=(\mu_1\mu_2\cdots\mu_v)^{\frac{1}{v}}$ be the geometric average of the true expression levels $\mu_1,\mu_2,\cdots,\mu_v$ of the v samples, then

$$Y=\gamma+\log_2\left(\mu_k/\bar{\mu}\right)-\log_2\left(\mu_{k'}/\bar{\mu}\right)+\varepsilon \equiv \gamma+\lambda_k-\lambda_{k'}+\varepsilon$$

where parameters $\lambda_k \equiv \log_2\left(\mu_k / \mu\right)$, k = 1, 2, … , v, represent the relative expression levels among v samples. It is easy to see that $\sum_{k=1}^{v}\lambda_k=0$. Among them, there are only v-1 independent parameters $\lambda_1,\lambda_2,\cdots,\lambda_{v-1}$ and $\lambda_v=-(\lambda_1+\lambda_2+\cdots+\lambda_{v-1})$.

For each gene, let $\underset{\sim}{Y}=(Y_1, \cdots, Y_n)^t$ denote the vector of the n normalization–corrected log-ratios obtained from all of the corresponding spots on slides in the experiment. In this experiment, n depends on gene and ranges between 16 to 64. The ordered set of the independent parameters is given by the parameter vector $\underset{\sim}{\beta}=(\lambda_1, \cdots, \lambda_{v-1},\gamma)^t$. Therefore, the data can be expressed as a linear model

$$\underset{\sim}{Y}=X\underset{\sim}{\beta}+\underset{\sim}{\varepsilon},\quad \underset{\sim}{\varepsilon}\sim N(0,\sigma^2 I)$$

where $\underset{\sim}{\varepsilon}=(\varepsilon_1, \varepsilon_2, \cdots, \varepsilon_n)^t$ is the vector of independent errors, I is the n×n identity matrix, and $X=(\underset{\sim}{x}_1, \underset{\sim}{x}_2, \cdots, \underset{\sim}{x}_n)^t$ is the n×v design matrix describing how the samples are paired onto slides. Each row of X corresponds to a spot. For instance, to a spot on the slide in which samples k ($\neq$v, labelled with Cy5) and k' ($\neq$v, labelled with Cy3) are mixed and hybridized, the corresponding row is

$$\underset{\sim}{x}^t=(\underbrace{0, \cdots, 0, \overset{\text{k-th}}{1}, 0, \cdots, 0, \overset{\text{k'-th}}{-1}, 0, \cdots, 0}_{\text{v-1}}, 1).$$

To a spot on the slide in which sample k ($\neq$v, labelled with Cy5/ Cy3) and sample v (labelled with Cy3/ Cy5) are mixed and hybridized, the corresponding row is of the following form:

$$
\begin{cases}
\underset{\sim}{x}^t = (\underbrace{1, \cdots, 1, \overbrace{2}^{\text{k-th}}, 1, \cdots, 1,}_{\text{v-1}} \; 1), & \text{if sample k is labelled with Cy5,} \\
\underset{\sim}{x}^t = (\underbrace{-1, \cdots, -1, \overbrace{-2}^{\text{k-th}}, -1, \cdots, -1,}_{\text{v-1}} \; 1), & \text{if sample k is labelled with Cy3.}
\end{cases}
$$

For the experiment shown in **Figure 1B**, the design matrix X is

$$
\begin{bmatrix}
1 & -1 & 0 & 0 & 0 & 0 & 0 & 0 & 0 & 0 & 0 & 1 \\
0 & 1 & -1 & 0 & 0 & 0 & 0 & 0 & 0 & 0 & 0 & 1 \\
0 & 0 & 1 & -1 & 0 & 0 & 0 & 0 & 0 & 0 & 0 & 1 \\
0 & 0 & 0 & 1 & -1 & 0 & 0 & 0 & 0 & 0 & 0 & 1 \\
0 & 0 & 0 & 0 & 1 & -1 & 0 & 0 & 0 & 0 & 0 & 1 \\
-1 & 0 & 0 & 0 & 0 & 1 & 0 & 0 & 0 & 0 & 0 & 1 \\
0 & -1 & 0 & 0 & 1 & 0 & 0 & 0 & 0 & 0 & 0 & 1 \\
0 & 0 & 1 & 0 & 0 & -1 & 0 & 0 & 0 & 0 & 0 & 1 \\
-1 & 0 & 0 & 0 & 0 & 0 & 1 & 0 & 0 & 0 & 0 & 1 \\
0 & -1 & 0 & 0 & 0 & 0 & 0 & 1 & 0 & 0 & 0 & 1 \\
0 & 0 & -1 & 0 & 0 & 0 & 0 & 0 & 1 & 0 & 0 & 1 \\
0 & 0 & 0 & -1 & 0 & 0 & 0 & 0 & 0 & 1 & 0 & 1 \\
0 & 0 & 0 & 0 & -1 & 0 & 0 & 0 & 0 & 0 & 1 & 1 \\
-1 & -1 & -1 & -1 & -1 & -2 & -1 & -1 & -1 & -1 & -1 & 1 \\
1 & 1 & 1 & 1 & 1 & 2 & 1 & 1 & 1 & 1 & 1 & 1 \\
0 & 0 & -1 & 0 & 0 & 0 & 0 & 0 & 1 & 0 & 0 & 1
\end{bmatrix}.
$$

Here we assume a situation in which there is a single replicate for each gene on each slide. If the number of replicates for each gene is greater than one, the corresponding row should be repeated accordingly.

The vector $\underset{\sim}{\beta}$ of unknown parameters can be estimated by the least square estimator $\hat{\underset{\sim}{\beta}}$, $\hat{\underset{\sim}{\beta}} = (X^tX)^{-1} X^t \underset{\sim}{Y}$ .The expression profiles presented in this study are $x_1 = \hat{\lambda}_1 - \hat{\lambda}_7$, $x_2 = \hat{\lambda}_2 - \hat{\lambda}_8$, $x_3 = \hat{\lambda}_3 - \hat{\lambda}_9$, $x_4 = \hat{\lambda}_4 - \hat{\lambda}_{10}$, $x_5 = \hat{\lambda}_5 - \hat{\lambda}_{11}$, and $x_6 = \hat{\lambda}_6 - \hat{\lambda}_{12}$.

Criterion to eliminate low variant genes (0.21):

To tease out gene having characteristic steady-state expression patterns, we filtered out all the genes with low variation avoiding the insignificant random noise. We used top 10 % variant genes as the candidates for further model fitting algorithm and the cutting value located at 0.21. (Text S1 Figure 1)

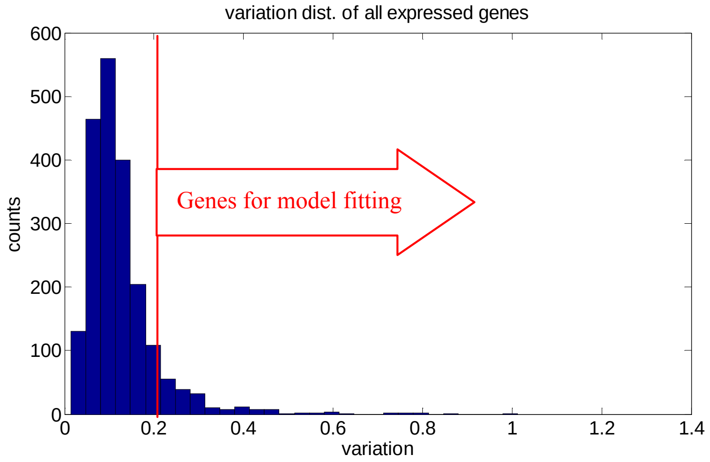

Text S1 Figure 1. The historgram of variation of each gene over 6 time periods. The cutting vale, 0.21 was set to filter out genes with low variation.

Criterion of significantly correlated to fitting model (0.75):

The null hypothesis of gene expression was set as $X = N(0,1)$. The distribution of correlation coefficient to the model (equation 2 in the main text) was shown as Text S1 Figure 2 (red line).

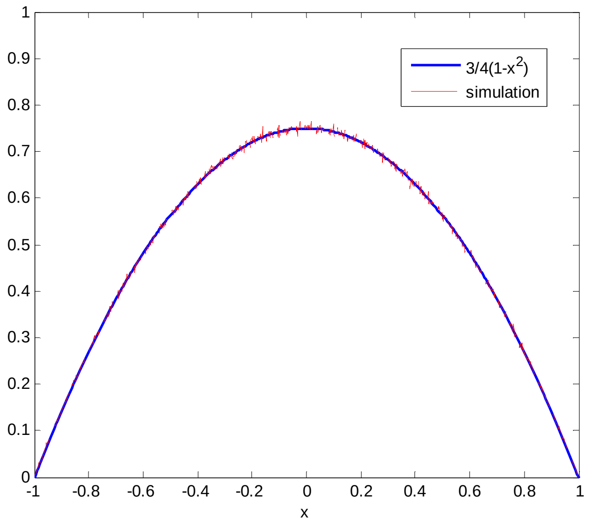

Text S1 Figure 2. The one-million-times simulation of the null hypothesis was shown as red line. Meanwhile, a PDF was shown in blue to describe the distribution of this simulation to gain the power of analytical results.

A density function, $f(x) = \frac{3}{4}(1 - x^2)$ was used to describe this distribution as shown in Text S1 Figure 2 (blue line).

Consequently, *p*-value(*p*) was determined by equation $p = \frac{1}{4}c^3 - \frac{3}{4}c + \frac{1}{2}$, where c is the cutting value(criterion). Regarding this work, the criterion we used to determine significantly correlated to fitting model, 0.75, corresponds to a *p*-value, 0.043.

The designate *p*-value was strongly case dependent. The strength of perturbation, the susceptibility of the reacting biosystems (transcriptomics in this paper), and the noise level all affect the suitable *p*-value. With this model, one can easily determine the cutting value and its corresponding *p*-value.
